# Epstein–Barr virus-acquired immunodeficiency in myalgic encephalomyelitis—Is it present in long COVID?

**DOI:** 10.1186/s12967-023-04515-7

**Published:** 2023-09-17

**Authors:** Manuel Ruiz-Pablos, Bruno Paiva, Aintzane Zabaleta

**Affiliations:** 1 Universidad Complutense de Madrid, Madrid, Spain; 2 Clinica Universidad de Navarra, Centro de Investigación Médica Aplicada (CIMA), IdiSNA, Instituto de Investigación Sanitaria de Navarra, Av. Pío XII 55, 31008 Pamplona, Spain

**Keywords:** Chronic fatigue syndrome, Myalgic encephalomyelitis, Long COVID syndrome, EBV EBNA-1, Post-acute COVID-19 syndrome, Immunodeficiency, HLA-II alleles, Inflammation

## Abstract

Both myalgic encephalomyelitis or chronic fatigue syndrome (ME/CFS) and long COVID (LC) are characterized by similar immunological alterations, persistence of chronic viral infection, autoimmunity, chronic inflammatory state, viral reactivation, hypocortisolism, and microclot formation. They also present with similar symptoms such as asthenia, exercise intolerance, sleep disorders, cognitive dysfunction, and neurological and gastrointestinal complaints. In addition, both pathologies present Epstein–Barr virus (EBV) reactivation, indicating the possibility of this virus being the link between both pathologies. Therefore, we propose that latency and recurrent EBV reactivation could generate an acquired immunodeficiency syndrome in three steps: first, an acquired EBV immunodeficiency develops in individuals with “weak” EBV HLA-II haplotypes, which prevents the control of latency I cells. Second, ectopic lymphoid structures with EBV latency form in different tissues (including the CNS), promoting inflammatory responses and further impairment of cell-mediated immunity. Finally, immune exhaustion occurs due to chronic exposure to viral antigens, with consolidation of the disease. In the case of LC, prior to the first step, there is the possibility of previous SARS-CoV-2 infection in individuals with “weak” HLA-II haplotypes against this virus and/or EBV.

## Introduction

Myalgic encephalomyelitis or chronic fatigue syndrome (ME/CFS) is a chronic, multisystem disease characterized by unexplained, disabling fatigue and a combination of nonspecific symptoms lasting at least 6 months [1, 2]. The main symptoms are fatigue, post-exertional malaise, cognitive dysfunction, sleep disturbances, generalized pain, and gastrointestinal discomfort [3–5]. The prevalence of patients with ME/CFS in the USA is estimated to be between 1.7 million and 3.38 million [6], corresponding to a range from 0.1 to 2.5% in the general population, depending on the diagnostic criteria applied [7]. The main causes are latent herpesvirus infection [2, 8–34], a chronic inflammatory state [1, 35–46], hypocortisolism [35, 47–60], autoimmune antibodies [13, 20, 24, 29, 35, 61–77], genetic predisposition [8, 35], or microclot formation [33, 78, 79].

SARS-CoV-2 infection, which causes a multisystem disease known as COVID-19 [80], is responsible for a worldwide pandemic with substantial mortality and morbidity [81]. Of all COVID-19 cases, approximately 81% have mild disease, 14% severe disease, and 5% develop critical illness [82]. It has recently been observed that approximately half of recovered COVID-19 patients continue to experience at least one long-term symptom [83, 84]. Within this group, long-lasting fatigue is particularly common, affecting 28.3% of patients [83]. The most common persistent symptoms are asthenia, dyspnea, pain, exercise intolerance, sleep disturbances, smell disturbance, taste disturbance, cognitive dysfunction, and neurological and digestive disturbances [5, 85–89]. Several terms, such as long COVID [89, 90], long-haul COVID-19 [91, 92], post-COVID syndrome [93], and post-acute sequelae of SARS-CoV-2 infection (PASC) [94], are used to refer to the persistence of these symptoms more than 12 weeks after SARS-CoV-2 infection [95, 96]. Although its origin is still unknown, among the main causes related to long COVID (LC) are the persistence of chronic SARS-CoV-2 infection [33, 97–105], autoimmunity [106–116], chronic inflammatory state with immune alteration [110, 117–127], Epstein–Barr virus reactivation [128–140], hypocortisolism [128, 133, 141–143], or microclot formation [33, 144–149].

The similarity in both symptoms and the different causes of ME/CFS and long COVID (LC) have led to searches for some connection between these two diseases [5, 33, 124, 139, 150–159]. The results of these studies suggest a high degree of similarity between LC and ME/CFS. Of the 29 symptoms observed in patients with ME/CFS, 25 were reported in patients with LC [156]. In addition, there are several articles that have linked EBV reactivations to the development of LC [138, 160–162]. Therefore, given the relationship of EBV with both diseases [2, 8–18, 20–28, 30–33, 128–130, 133–140], we propose that latency and recurrent EBV reactivation could generate both an acquired immunodeficiency syndrome in genetically predisposed individuals ME/CFS patients and, second, immunosuppression due to SARS-CoV-2 in the case of LC patients.

It should be mentioned that not only EBV infection has been linked to ME/CFS, but also human herpesvirus (HHV)-6, cytomegalovirus (CMV), human parvovirus B19, and enteroviruses [22, 23, 28, 163–168], as well as bacterial and parasitic infections [169]. However, as in the case of LC, in these other post-infectious subgroups of ME/CFS, EBV could also play a possible role in immunological alterations and symptomatology, since the virus is present in 90% of the population [170] and therefore could be reactivated in the presence of immunosuppression [128–130, 133–140].

## Common model of EBV-acquired immunodeficiency

Epstein–Barr virus (EBV) is known to be involved in the development of some autoimmune diseases and certain cancer and is suspected to underlie the development of chronic fatigue syndrome/myalgic encephalomyelitis and LC [2, 8, 23, 26, 33, 129, 133, 134, 136–138, 171–182]. However, the pathways it exploits leading to the development of these diseases remain unclear, although it is thought to be mainly related to the genetic susceptibility of an individual [171].

**Fig. 1:**
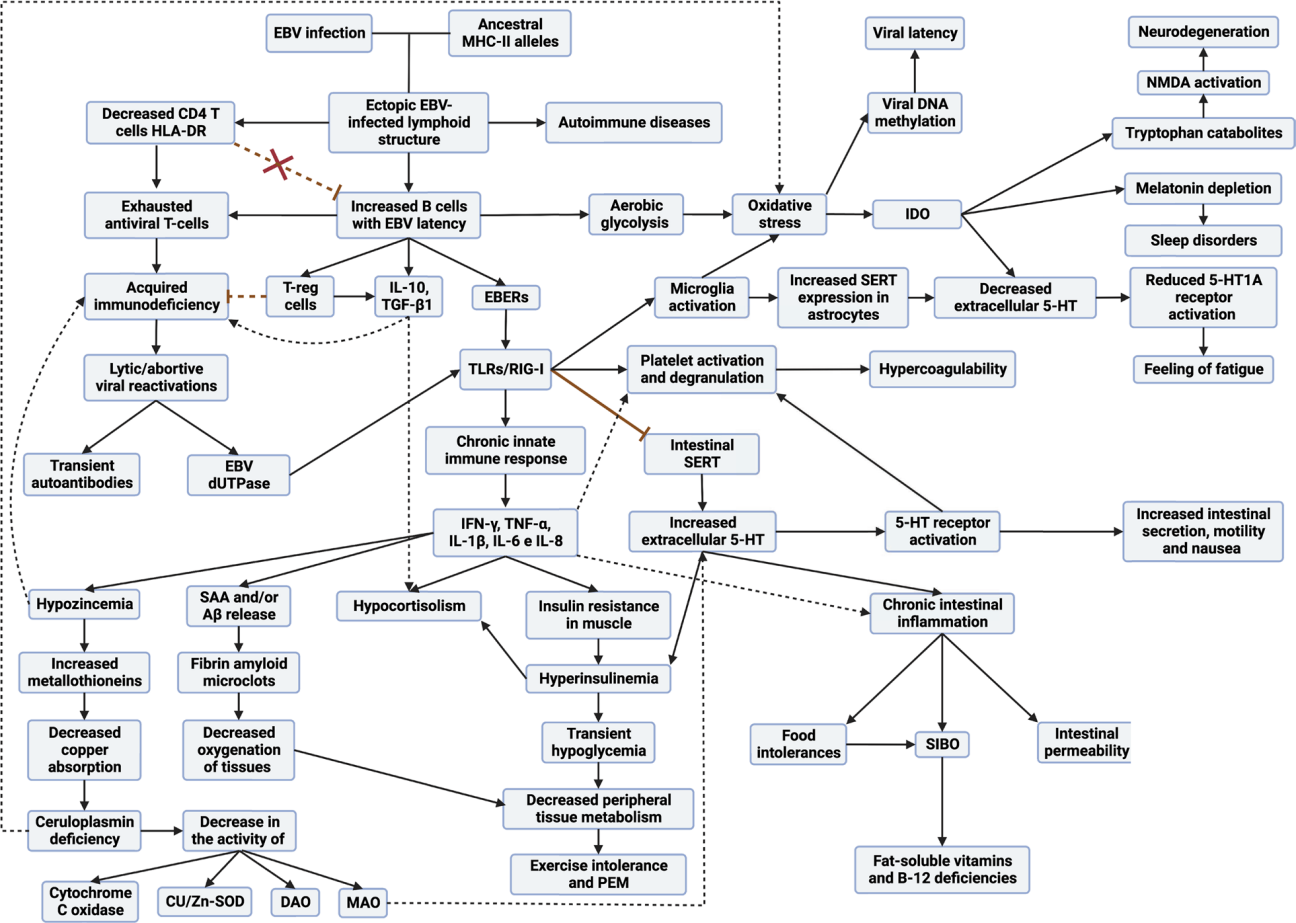
Schematic model of the development of acquired immunodeficiency following EBV infection

**Fig. 2:**
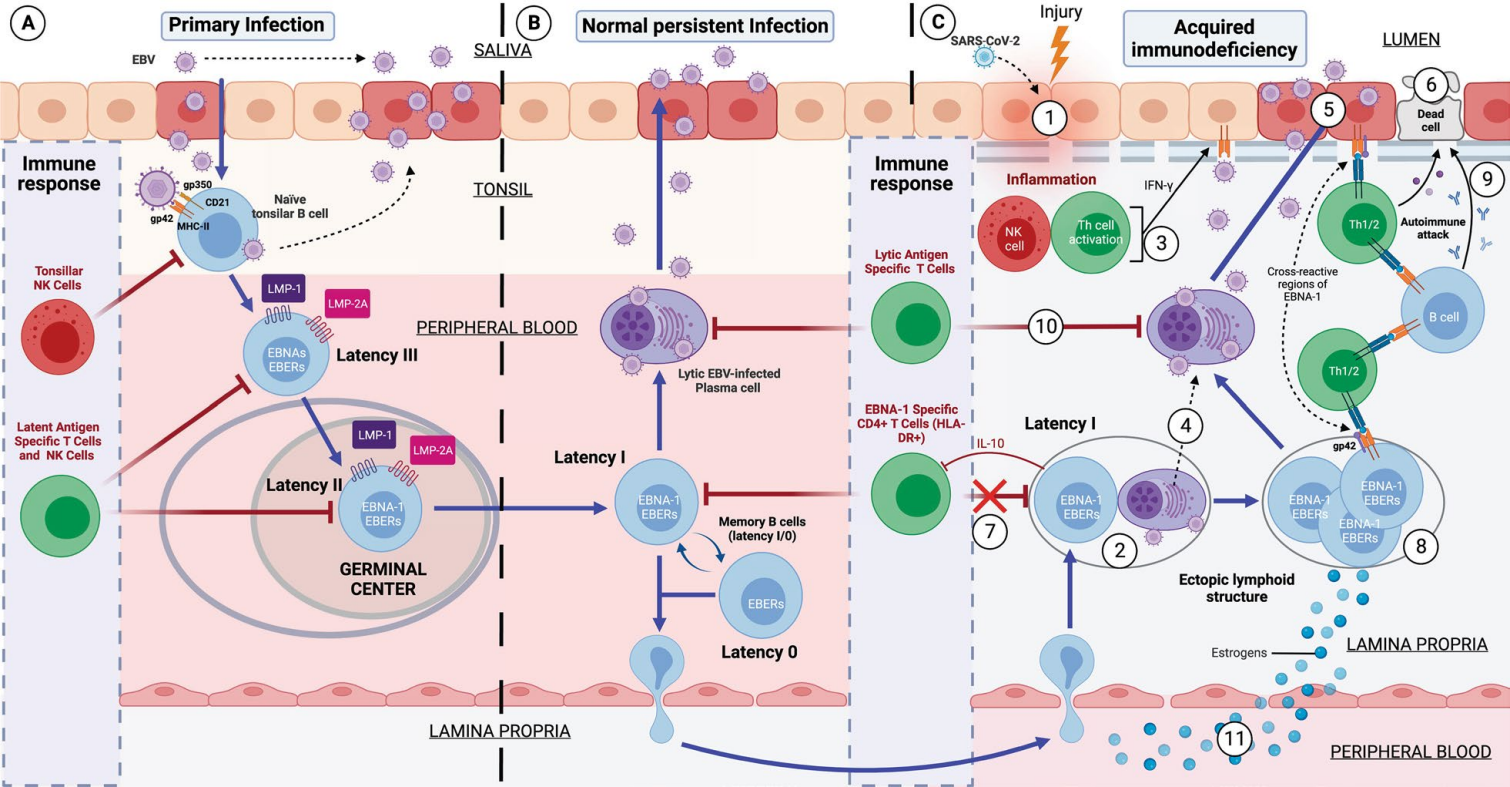
Development of Epstein–Barr Virus (EBV)-induced acquired immunodeficiency in patients with genetic susceptibility. **A** Primary infection by EBV. EBV is transferred to the host via the saliva of an infected carrier, initially infecting the epithelial cells of the pharynx and subsequently naïve B lymphocytes in the tonsils, through interactions between the virus’s glycoproteins gp350 and gp42 and the host cells’ CD21 and Class II MHC (MHC-II) molecules, respectively. Lytic infection creates new viral particles that continue to infect more epithelial cells. Subsequently, the EBV-infected B cells enter a state of peripheral latency, during which they express a set of specific viral genes, including LMP1, LMP2A, EBNAs, and EBER (latency III). These latency III B cells progress through the germinal center reaction to latency II and emerge as memory B cells with latencies I/0 that establish a lifelong latent infection (**B**). The healthy host’s immune response is sufficient to control the EBV infection. NK cells in tonsil produce high levels of IFN-γ that withhold the transformation of B cells by EBV during earlier stages of the infection. Type III and II latency B cells are regulated by NK and T cells specific for latent proteins. However, memory B cells with type I latency are only controlled by activated EBNA-1 specific CD4 T cells. EBV-infected plasma cells can periodically enter in lytic phase, but are controlled by CD4 and CD8 T cells with specificity for EBV lytic proteins. **C** Development of EBV-induced acquired immunodeficiency and autoimmunity in the mucosa of genetically susceptible patients. (1) An inflammatory stimulus or other infection (SARS-CoV-2) recruit leukocytes in the mucosa, including latency I (EBNA-1) B cells and healthy B cells. (2) In the mucosa, B cells form ectopic lymphoid aggregates that enable the generation of antigen-specific immune responses. These ectopic lymphoid structures create a favorable environment for the transformation of EBV latent B cells into proliferating blasts, to become memory B cells. (3) Furthermore, activation of NK cells occurs in response to both the initial inflammatory stimulus and to restrict B cell transformation by EBV. Exposure to foreign antigens from the initial stimulus or to viral antigens from EBV leads to the activation of CD4 T cells and the release of IFN-γ, followed by upregulation of MHC-II on epithelial cells, promoting the acquisition of a non-professional antigen-presenting cell phenotype. (4) Additionally, the presence of foreign antigens could also trigger terminal differentiation and activation of latent EBV B lymphocytes, allowing the transition from the latent phase to the lytic virus phase. (5) Subsequently, newly generated viral particles infect more epithelial cells through gp42/MHC-II interaction, leading to increased inflammation and ultimately to latent EBV infection. Moreover, this chronic inflammation induces a cytokine response, leading to further recruitment of B cells and perpetuation of viral infection. (6) Latent EBV epithelial cells could enter a lytic phase, releasing new virions, undergoing lysis due to T cell response, or experiencing neoplastic transformation. (7) Immune evasion mechanisms of EBV latency (epithelial and B cells) involve a decrease in activation and cytotoxic capability of EBNA-1-specific CD4 T cells through the release of IL-10 and EBV miRNAs contained in exosomes, which could suppress the expression of target genes in the viral or host genome to maintain latent EBV infection. (8) This altered immunosurveillance leads to increased proliferation of EBV-latent B- and epithelial cells, which raises the risk of neoplastic transformation or autoimmune disease in genetically predisposed patients with ancestral MHC-II alleles susceptible to EBV. (9) The presentation of native cellular autoantigens or viral EBNA-1 through MHC-II/gp42, which can undergo post-translational modifications, such as citrullination, and form neoantigens, could trigger the activation of autoreactive CD4 T cells and the formation of autoantibodies against tissue cells. (10) Other virus latency phases or the lytic phase would be controlled by NK and CD4 and CD8 T cells, specific for the EBV lytic proteins. (11) In women, estrogens elevate the risk of developing EBV-induced acquired immunodeficiency by reducing the CD4/CD8 T lymphocyte ratio, increasing the survival of B cells, promoting the release of antibodies, and increasing the expression of the major histocompatibility complex class II

HLA-II genes are highly polymorphic due to coexistence with different pathogens as humans moved to different regions, leading to the emergence of new alleles providing resistance to these pathogens [183, 184]. DR2-DQ6, DR3-DQ2, and DR4-DQ8 are among the oldest extant haplotypes [185]. These ancestral haplotypes have survived over time by being able to recognize a greater number of antigens than other haplotypes, enabling them to detect and eliminate a greater number of pathogens [183]. For example, HLA-DR4 alleles are associated with increased clearance of hepatitis B virus infection [186], and HLA-DRB1*0401 or DRB1*1501 alleles are associated with increased clearance of hepatitis C virus infection [187]. Surviving a greater number of pathogens has allowed these ancestral haplotypes to be inherited from generation to generation, but with the disadvantage that those older pathogens with which they have coexisted have also acquired resistance against them via co-evolution of their evasion mechanisms [171, 183]. One of these pathogens is EBV, which is the only member of the genus *Lymphocryptovirus* adapted to humans and was transferred to a hominid ancestor millions of years ago [188]. Its gp42 protein is an evasion mechanism that has evolved against these ancestral haplotypes, as the virus infects cells through the interaction of its viral protein gp42 with the β1 domain of HLA-II in the cell [189–191]. In this way, its membrane fuses with that of the cell, forming a new gp42–HLA-II complex that alters antigenic presentation to CD4 T cells (Fig. 1) in individuals carrying these alleles [189, 190, 192], managing to suppress the activation (HLA-DR) of CD4 T cells and the presentation of EBV nuclear antigen (EBNA)-1 on HLA-II molecules [171]. Not presenting EBNA-1 to CD4 T cells via HLA-II allows these cells with latency I to forgo elimination, since CD4 T cells are the only ones capable of recognizing this latency [171, 193]. This allows any inflammatory stimulus (such as another infection) in any tissue to recruit leukocytes (among which are cells with EBV latency), ultimately leading to the formation of EBV-infected ectopic lymphoid structures (Fig. 2) [171–176, 194–196]. When unrecognized, they proliferate in these ectopic lymphoid structures, triggering the activation and release of IFN-γ by NK cells to restrict the transformation of B cells by the virus and as a response to the first inflammatory stimulus [197, 198]. In addition, the presence of foreign antigens from the first inflammatory stimulus leads to terminal differentiation and activation of EBV latent B cells, allowing the transition from the latent to the lytic phase of the virus, originating foci of viral reactivation [171, 172, 194]. Therefore, exposure to foreign antigens from the first stimulus or to EBV viral antigens leads to activation of CD4 T cells and IFN-γ release, provided that the cells presenting these antigens to CD4 T cells are not previously infected with EBV, since the gp42–HLA-II complex in these cells would prevent their activation [171, 194]. Both IFN-γ released by NKs and CD4 T cells upregulates HLA-II expression in adjacent cells (such as epithelial cells), which favors the acquisition of a nonprofessional antigen-presenting cell phenotype [199, 200]. The increased expression of HLA-II in these cells allows the newly generated viral particles to infect more cells through the gp42–MHC-II interaction, which ultimately leads to an increased number of latent cells that perpetuate the inflammatory state in that tissue by releasing proinflammatory substances (Fig. 1), such as Epstein–Barr virus-encoded small RNAs (EBERs) [201–203], producing abortive reactivation, in which the resulting proteins can cause inflammation [204, 205] or induce the release of new virions that provoke an immune response [171]. This chronic inflammation provokes a chemokine response, leading to an increase in the recruitment of a greater number of B cells, allowing the perpetuation of viral infection and, ultimately, the formation of EBV reservoirs in these tissues [171, 194]. Increasing the number of EBV-dormant cells would also increase the evasion mechanisms of these cells by decreasing the activation and cytotoxic capacity of EBNA-1-specific CD4 T cells through the release of IL-10 (increased humoral Th2 response), chemotaxis of regulatory T lymphocytes (Treg) due to increased CCL20 expression in EBNA-1-mediated EBV latency cells, and release of EBV miRNA contained in exosomes, which could suppress the expression of target genes in the viral or host genome to maintain latent EBV infection [206–208]. Consequently, the greater the number of antigen-presenting cells infected by this virus, the lower the activation of CD4 T cells, either by inhibition of the TCR–HLA-II interaction by the gp42–HLA-II complex or due to the evasion mechanisms of these latently infected cells. Thus, an acquired immunodeficiency in CD4 T-cell function develops, leading to increased proliferation of latent EBV cells, which increases the risk of neoplastic transformation or autoimmune disease in these tissues [171].

## Sex differences in immunodeficiency

**Fig. 3:**
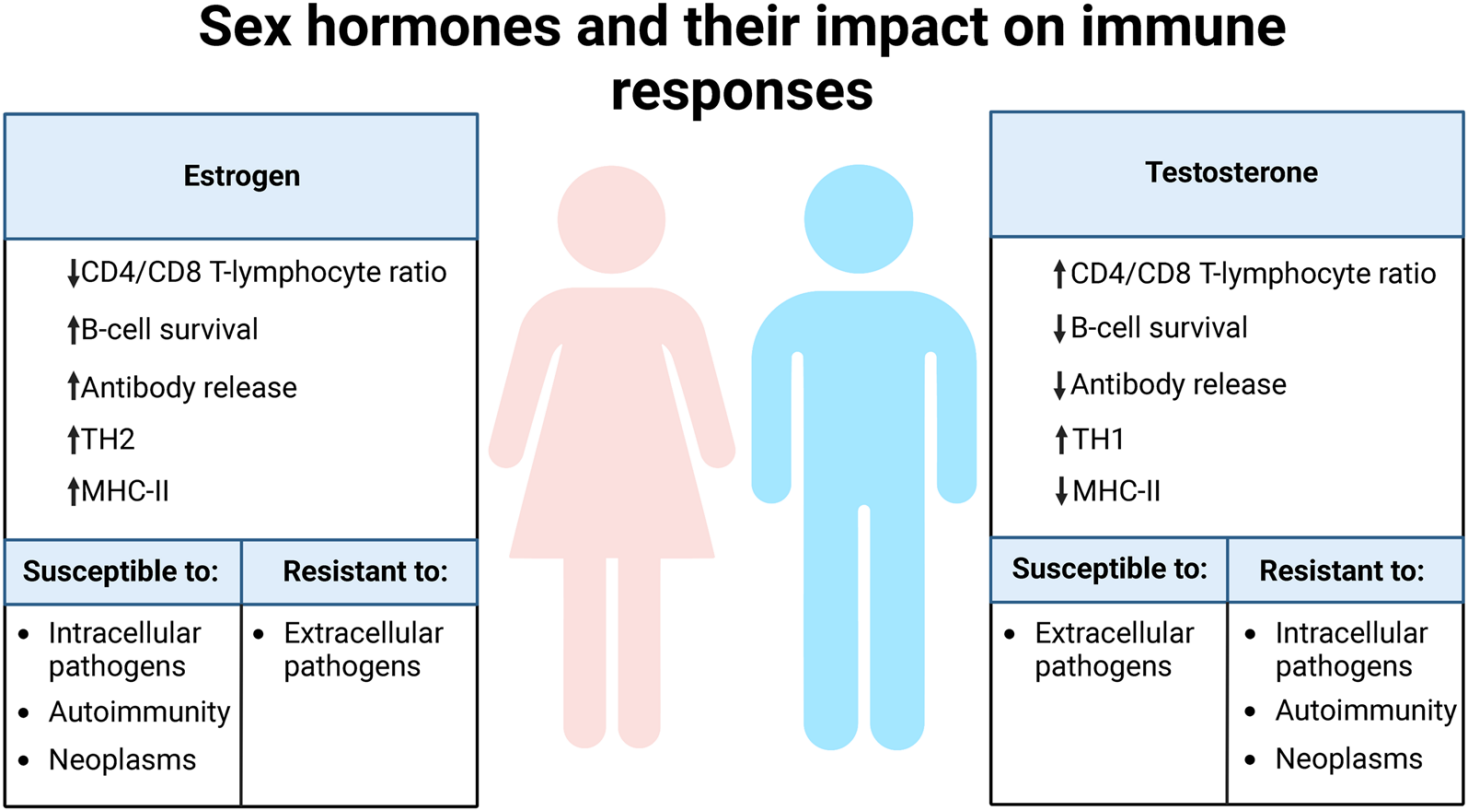
Sex hormones and their impact on immune responses. This figure provides an overview of the influences of estrogens and testosterone on immune responses, highlighting their effects on susceptibility and resistance to various conditions and pathogens

The characteristics of this acquired immunodeficiency could vary according to biological sex (Fig. 3). In fact, estrogens decrease the CD4/CD8 T-lymphocyte ratio and increase B-cell survival, antibody release, and expression of the class II major histocompatibility complex [209–211]. In women, this increase in antibody levels would increase their resistance to viral infections under normal conditions, but under pathological conditions there is increased B cell survival, decreased CD4 T cells, and increased estrogen-induced HLA-II expression, which confer certain disadvantages against EBV, as they increase the survival of EBV-transformed B cells, and the increase in HLA-II expression allows more cells to become infected [171, 209]. In addition, increased HLA-II expression can lead to increased presentation of native cellular autoantigens or viral antigens, which can undergo posttranslational modifications, such as citrullination, and form neoantigens, through the endogenous antigen processing pathway, triggering the activation of autoreactive cells [171, 212–215]. Therefore, both the increase in the survival of transformed B cells and the increase in antigen presentation generated by the increase in HLA-II expression by estradiol may favor an increase in the presentation of both self and foreign antigens during an infectious process, and those “abnormal” plasma cells producing autoantibodies survive longer, which consequently increases the probability of women developing autoimmune diseases or even cancer [171]. In the case of androgens, testosterone regulates the immune system by increasing the Th1 response and CD8 T cell activation while downregulating the NK cell response and HLA-II expression [209] . As antigen-presenting cells are important for the differentiation of CD4 or helper T cells into Th1 or Th2 cells, depending on the cytokines they release, sex hormones could determine whether CD4 T cells differentiate more into Th1 or into Th2 [209]. Thus, women have a higher incidence of sex-related diseases but generate a more effective response against extracellular infections by having more antibodies (Th2 response) [209]. The female immune system might however be more ineffective against intracellular pathogens than in the male immune system, as more cellular (Th1) response is needed to eliminate virus-infected cells [209].

In addition to these considerations, the menstrual cycle in women may also affect acquired immunodeficiency [216]. During the cycle, elevated estrogen and progesterone levels induce Th2-type responses, whereas low estrogen concentrations induce Th1-type responses [216]. In addition, progesterone suppresses Th1-type responses by stimulating regulatory T cells and increasing Th2-type responses [216]. Therefore, there is a predominantly Th1 response during the beginning of the follicular phase, the end of the luteal phase, and menstruation, since estrogen and progesterone levels are not elevated [216]. In contrast, during the late follicular phase, ovulation, and in the luteal phase, there is a predominantly Th2 environment, which causes some immunosuppression [216]. For this reason, during the phases with a predominant Th2 response, greater immunosuppression can appear, causing outbreaks of viral reactivation as occurs, for example, with the herpes simplex virus type 1, which in these phases usually causes vesicles to appear on the lips [217]. Therefore, if there is a previous immunodeficiency, it would worsen during these phases.

## Consequences of EBV-acquired immune deficiency

### Development of autoimmune diseases

Since EBV was transferred to a hominid ancestor millions of years ago, and as the DRB1*04, *03 and *02 lineages are the oldest [185], it may be thought that those individuals with the DR2-DQ6, DR3-DQ2, or DR4-DQ8 haplotypes—against which the immune evasion mechanisms of this virus have evolved the most—may be less resistant to infection, and have a higher risk of developing EBV-associated diseases [190, 191, 218]. These ancestral haplotypes, which contain HLA-II alleles that bind EBV epitopes suboptimally [190, 191, 218], are the main ones linked to the development of autoimmune diseases, but not all individuals carrying these haplotypes develop these diseases, indicating that they are caused by an environmental factor—infection [183]. EBV is the main suspect, as it is closely related to the alleles of the β1 domain of HLA-II [8, 171] and could explain why EBV-associated autoimmune diseases are related to the β DRB1* and DQB1* alleles [190, 191, 218]. In addition, glutamic acid 46 (E46) and arginine 72 (R72) of HLA class II are essential for a stable interaction between Gp2 and MHC-II, where R72 is conserved in all HLA-DR,-DP alleles, but E46 is conserved in all HLA-DR,-DP alleles and only in a small subset of HLA-DQ alleles: β * 02 (β * 0201, β * 0202, and β * 0203) [219–221]. This suggests that individuals with HLA-DQ β * 02 alleles may have an increased susceptibility to infection in tissues with unique HLA-DQ expression [221].

Therefore, depending on the tissue infiltrated by the B cells with EBV latency in an individual with EBV “weak” HLA-II alleles, the formation of EBV-infected ectopic lymphoid structures and an infection of other cell types by the increased expression of HLA-II as a result of increased IFN-γ, one type or another of EBV-associated disease may develop [171–176, 195]. Presentation through MHC-II of native cellular autoantigens or viral EBNA-1 (Fig. 2), which can undergo posttranslational modifications, such as citrullination, and form neoantigens, could trigger the activation of autoreactive CD4 T cells and the formation of autoantibodies against tissue cells [171, 212–215]. EBNA-1 exhibits molecular mimicry with self-antigens [212, 222–226], generating IgG against these self-proteins, and/or a specific cellular response.

### Chronic response of innate immunity due to loss of adaptive functions

Increased proliferation of latency I B cells as a result of low recognition by EBNA-1-specific cytotoxic CD4 T cells could lead to an increase in latency II and III and lytic phase cells. This leads to the continuous activity of NK cells and T cells (CD4 and CD8) with specificity for EBV latent and lytic proteins [198]. Therefore, lack of control of latency I cells leads to persistent infection, where chronic exposure to these viral antigens could lead to a “depletion” of antiviral T cells, as occurs in chronic infections where the individual fails to generate a robust antiviral response [227]. In chronic viral infections, there is usually an increase in the generation of short-lived effector cells and exhausted T cells, decreasing the formation and function of memory T cells, which are those most needed to control future exposures to the same virus and latencies to other viruses [171, 228]. These depleted antiviral T cells (PD-1+/Tim-3+) are functionally ineffective, as they are unable to activate and exert their cytotoxic functions in response to antigenic stimulation [227, 229], thus allowing the infection to become even more chronic. In our model, T-cell depletion is preceded by a failure of CD4 T-cell activation and response, as CD4 T cells are essential for maintaining CD8 T-cell cytotoxic responses [230]. Therefore, chronic antigenic stimulation by EBV in the absence of CD4 T-cell support results in a complete loss of antiviral adaptive functions in infected tissues [230].

Since adaptive immunity fails to control viral infection, a continuous innate immune response is generated. EBERs (EBER1 and EBER2) are noncoding double-stranded RNAs released by EBV latency cells in these ectopic lymphoid structures and are the main molecules that activate innate immunity through Toll-like receptors 3 (TLR3) and retinoic acid-inducible protein-1 RNA helicase (RIG-I) [231, 232]. Activation of these receptors results in some cells (such as epithelial cells or cells of the innate immune system) producing IL-32, a cytokine that activates/differentiates monocytes into macrophages and induces the production of proinflammatory cytokines such as IL-1, IL-6, and TNFα by activating NF-κB in differentiated macrophages [231–233]. This results in the recruitment of macrophages into infected tissues that aid in maintenance of the chronic inflammatory state by releasing these proinflammatory cytokines [231]. In addition, IL-32 is implicated in the maintenance of EBV latency by inactivating the activity of the Zta promoter, which is required to initiate the lytic phase [234]. In this way, it further evades adaptive immunity.

On the other hand, EBERs can also cause monocytes to become activated and differentiate into dendritic cells via activation of TLR3 receptors, which results in the release of high levels of IL-12 leading to activation of NK cells (CD16-CD56^bright^) [233]. Activation of NK cells limits B cell transformation and proliferation by releasing IFN-γ, which allows delayed expression of the latent membrane protein 1 (LMP1) on newly infected cells [233]. This restricts infection until adaptive immunity kicks in. In our model, however, there is acquired immunodeficiency, which compromises adaptive immunity, thus chronifying the innate immune response with continued release of proinflammatory cytokines (IL-6, TNFα, and IFN-γ) and preventing the infection from ever resolving.

IL-6 can be secreted both by monocytes activated by EBERs via TLR3 and by EBV-immortalized cells [233]. In the latter, it acts as an autocrine growth factor [233, 235]. It also favors the Th2 response of CD4 T cells by promoting IL-4 expression and by preventing CD4 T cells from responding to IFN-γ, thus impeding Th1 responses [236]. Therefore, increases in the level of IFN-γ released by NK cells do not favor the Th1 response of CD4 T cells due to increased IL-6 levels and the evasion mechanisms of latent cells that promote a Th2 response.

### Reactivation in the presence of transient autoantibodies

One of the consequences of the decrease in the activation and function of cytotoxic CD4 T cells is the loss of immunosurveillance of latent infections of other pathogens (Fig. 1) in the individual, since these cells are necessary to control cells with latency or in lytic phase of Parvovirus B19, EBV, cytomegalovirus, and other herpesviruses [237–239]. Thus, it will eventually lead to an increase in viral reactivation depending on the degree of immunodeficiency present in the individual [239]. This is supported by the fact that reactivation of latent pathogens increases during periods of increased immunodeficiency, which results in disease outbreaks throughout the year depending on whether they are reactivated or not, as occurs in systemic lupus erythematosus [240].

In addition, transient autoantibodies such as rheumatoid factor, antinuclear antibodies (ANA), antiphospholipid antibodies, and antineutrophil cytoplasmic antibodies (ANCA) may be generated during these viral reactivation, which disappear after infection control but may contribute to symptoms during disease flares [241–244]. In the case of ANCAs, they are not only produced during reactivation of these viruses but also by EBV latent B cells [245].

### Abortive lytic replications

Due to the loss of detection of EBV latency cells in immunosurveillance, abortive lytic replications may increase, releasing dUTPase proteins from EBV into the environment [10]. These dUTPases are only expressed during lytic replications or abortive lytic replications and can also increase the secretion of the proinflammatory cytokines (IL-1β, IL-6, IL-8, IL-12, TNF-α, and IFN-γ) and IL-10 (Fig. 1) by human dendritic cells, macrophages, and peripheral blood mononuclear cells (PBMC) via TLR2 activation [10, 246]. In this way, they also contribute to the chronic innate response and symptomatology.

### Insulin resistance and hyperinsulinemia

High levels of IFN-γ produced by NK cells in response to persistent infection cause insulin resistance in skeletal muscle by downregulating insulin receptor transcription in myocytes [247, 248] but without this occurring in the liver, as it does in type 2 diabetes mellitus [247, 249]. To compensate for insulin resistance in muscle and maintain euglycemia, the pancreas increases insulin production, causing compensatory hyperinsulinemia [247–249]. However, hyperinsulinemia can reduce glycogenolysis in the liver, leading to reduced production of glucose and decreased levels in peripheral blood (transient hypoglycemia), which promotes fasting metabolism [249]. Under normal conditions of infection, transient hypoglycemia is beneficial, as it amplifies the cellular stress response of infected cells, leading to increased production of antiviral cytokines such as type I interferons, thus impairing viral replication [249]. That is, the higher the glucose levels, the higher the replication rate. In immunocompetent patients, the occurrence of hyperinsulinemia serves to enhance the innate immune response and enhance the antiviral effector response of CD8 T cells, as insulin receptor and CD28 signaling converge on PI3K [247, 248]. However, EBV has evolved to evade this cytokine, as IFN-γ upregulates HLA-II expression in adjacent cells, allowing the virus to infect more cells through the gp42–MHC-II interaction [201–203]. Furthermore, in our model of acquired immunodeficiency in CD4 T-cell function, CD8 T cells are “exhausted” and do not respond well to stimuli without the help of CD4 T cells. Hence, hyperinsulinemia and transient hypoglycemia persist over the long term. Thus, a chronic state of hyperinsulinemia coupled with increased glucose consumption via aerobic glycolysis (Warburg effect) in EBV-transformed cells could cause recurrent transient hypoglycemia (Fig. 1), leading to various symptoms [250, 251].

### Autonomic and central nervous system symptoms

The consequences of chronic hyperinsulinemia are that individuals are more susceptible to adrenal crisis due to a deficient cortisol response to hypoglycemia or any physical or mental stress process [252, 253]. Recurrent transient hypoglycemia can generate different symptoms through neuroglucopenic or autonomic pathways [252]. Autonomic symptoms caused by sympathetic activation may include tachycardia, anxiety, tremors, sweating, nausea/vomiting, and hypothermia [252]. Symptoms at the central nervous system level include headaches, lethargy, chronic fatigue, neurocognitive dysfunction, and motor/sensory/visual disturbances, among others [252].

The inability of insulin to effectively stimulate glucose uptake in skeletal muscle due to insulin resistance could result in decreased physical performance [254]. In addition, both EBV-induced vascular damage and clogging of amyloid microcoaguli could reduce muscle blood flow and oxygen diffusion, which could also negatively affect skeletal muscle performance [147, 255, 256]. Even during physical exercise, there is increased release of proinflammatory cytokines such as IL-6 in skeletal muscle, worsening already existing inflammation and thus further exacerbating symptoms [257]. This release of proinflammatory cytokines is directly proportional to exercise intensity and duration [257]. Thus, muscle insulin resistance, reduced oxygen diffusion, and increased proinflammatory cytokines during physical exercise could lead to the development of exercise intolerance or post-exertional malaise, which is worse after aerobic exercise.

Vascular damage and microclot obstruction could also occur in other tissues where lack of oxygen transport could lead to organ dysfunction, postural tachycardia syndrome, muscle myalgia, neurological disorders, cognitive impairment, and lactic acidosis [147, 258]. This, together with increased oxidative stress, could lead to an increase in visual problems as a consequence of oxidative stress damage to the retina, as occurs for example in patients undergoing prolonged chronic treatment with chloroquine [259, 260].

Therefore, the variety of symptoms will depend on the degree of immunodeficiency present in the individual, the affected tissues, and outbreaks of viral reactivation. In the latter case, the increase in viral reactivation (especially of EBV and Parvovirus B19) or abortive reactivation during periods of increased immunodeficiency may contribute to the exacerbation of symptoms by increasing the production of transient autoantibodies such as ANCAs, causing symptoms of vasculitis [241], and by increasing the release of viral dUTPases, which increase neurological symptoms [246].

### Hypocortisolism

Under acute conditions the proinflammatory cytokines IFN-γ, TNFα, IL-1, and IL-6 stimulate the hypothalamic–pituitary–adrenal (HPA) axis by stimulating ACTH secretion [54, 236]. In this way, IFN-γ not only serves to activate macrophages but also allows for increased glucocorticoid receptor expression for subsequent feedback inhibition [236]. In addition, so that this inflammatory response is not exaggerated, there is stimulation of cortisol secretion due to the increase in ACTH, resulting from the action of IFN-γ and direct stimulation of the adrenal gland by IL-6 [236, 261]. During persistent infections, however, chronic exposure to IL-10, TGF-β1, and TNFα could suppress ACTH-stimulated cortisol secretion in the adrenal gland and thus cause relative hypocortisolism (Fig. 1) during chronic infections [54, 262, 263]. Both IL-10 and TGF-β1 (anti-inflammatory and immunosuppressive cytokines) are elevated in EBV infections, where they are secreted by EBV latency cells and regulatory T cells to counteract proinflammatory cytokines and to evade CD4 T cells [8, 264, 265]. Thus, the greater the number of infected tissues with EBV reservoirs, the greater the level of secretion of these anti-inflammatory cytokines and, thus, the greater the negative feedback on cortisol secretion. Disease progression and acquired immunodeficiency in CD4 T-cell function may however lead to an increase in the replicative rate of the virus and infection in the adrenal glands or pituitary gland, as occurs in AIDS by decreasing surveillance of herpesviruses (such as cytomegalovirus), allowing them to infect and deplete cortisol stores [236, 266]. Both cases would result in relative hypocortisolism, where the small amount of cortisol synthesized by negative feedback would bind to glucocorticoid receptors on the cells of innate immunity to suppress proinflammatory cytokine synthesis, though only partially due to the persistence of infection and greater positive feedback from proinflammatory cytokines [236, 267]. In addition, chronic high insulin levels may also be playing an important role in the development of hypocortisolism, as insulin can cross the blood–brain barrier and bind to insulin receptors in the hypothalamus, inhibiting the secretion of corticotropin-releasing hormone (along with others such as growth hormone) and, therefore, its downregulation would inhibit pituitary ACTH secretion [268–270]. This negative regulation of cortisol secretion can be observed, for example, in patients with insulinomas, where increased insulin causes a deficient cortisol response to hypoglycemia, whereas cortisol responses are normalized after their resection [253]. Both growth hormone and cortisol participate in glucose metabolism, increasing gluconeogenesis and antagonizing the effects of insulin [270]. In addition, they participate as counter-regulatory hormones during hypoglycemia [270]. Thus, it seems that in the face of persistent infection, the body tries to maintain a state of transient hypoglycemia caused by an increase in proinflammatory cytokines, hyperinsulinemia, and hypocortisolism in order to maintain a chronic innate immune response, decrease viral replication, and enhance the effector response of CD8 T cells. However, this normalized state is not achieved in individuals with “weak” HLA-II haplotypes against EBV, presenting an impaired CD4 T-cell function and, therefore, a deficient CD8 T-cell cytotoxic response.

### Alteration of the serotonergic system

Apart from the release of proinflammatory cytokines, the activation of TLR3, by EBERs, or of TLR2, by EBV dUTPases, also causes inhibition and decreased expression of serotonin transporters (SERT), leading to extracellular accumulation of serotonin (5-HT) in inflamed tissues (Fig. 1) [271–274]. For example, its inhibition in the digestive mucosa causes a reduction of SERT activity in enterocytes, thus decreasing 5-HT uptake and metabolization [271, 272]. This accumulation of extracellular 5-HT causes the activation of 5-HT3 receptors of enterochromaffin cells, stimulating increased release of 5-HT [275]. This increase in extracellular 5-HT in the intestinal mucosa activates the 5-HT receptors (such as 5-HT3) of myenteric neurons, smooth muscle cells, and epithelial cells, which causes increased intestinal secretion and motility to aid in pathogen clearance [272, 276, 277]. In addition, vagal responses are elicited by activating 5-HT3 afferent receptors, which induces nausea and vomiting [278]. Another type of cell involved in 5-HT elevation in the digestive mucosa or in any inflamed tissue is platelets. Under normal conditions, platelets collect excess 5-HT from the intestinal mucosa through SERT on their surface, preventing it from accumulating and contributing to the termination of its enteric activity [276]. They then transport it to other tissues, such as liver and bone [276]. Under inflammatory conditions, 5-HT binds to the 5-HT2A receptors on platelets, due to their increase in inflamed tissue, which causes their activation and degranulation, releasing more 5-HT into such tissue [271, 279]. In addition, binding of EBERs to the platelet TLR3 receptor causes a decrease in SERT activity and thus platelet 5-HT reuptake [279, 280]. Moreover, chronic increases in plasma 5-HT levels also lead to a reduction in SERT expression, further decreasing its uptake by platelets [281]. Therefore, under pathological conditions, there is excess release of 5-HT, saturation of its metabolic pathways, and decreased transport, leading to an increase in its levels in peripheral blood and provoking different responses. This process persists until adaptive immunity resolves the infection, allowing everything to return to normal.

Increased levels of 5-HT in peripheral blood could activate pancreatic β-cells and increase their proliferation through the 5-HT3 receptor, leading to increased release of insulin and 5-HT by these cells [282]. This 5-HT released by β-cells activates the 5-HT1F receptors of neighboring α-cells, leading to reduced cAMP levels and inhibition of glucagon secretion [283]. Consequently, 5-HT is also involved in the maintenance of a hyperinsulinemic state with transient hypoglycemia during chronic infections.

**Fig. 4:**
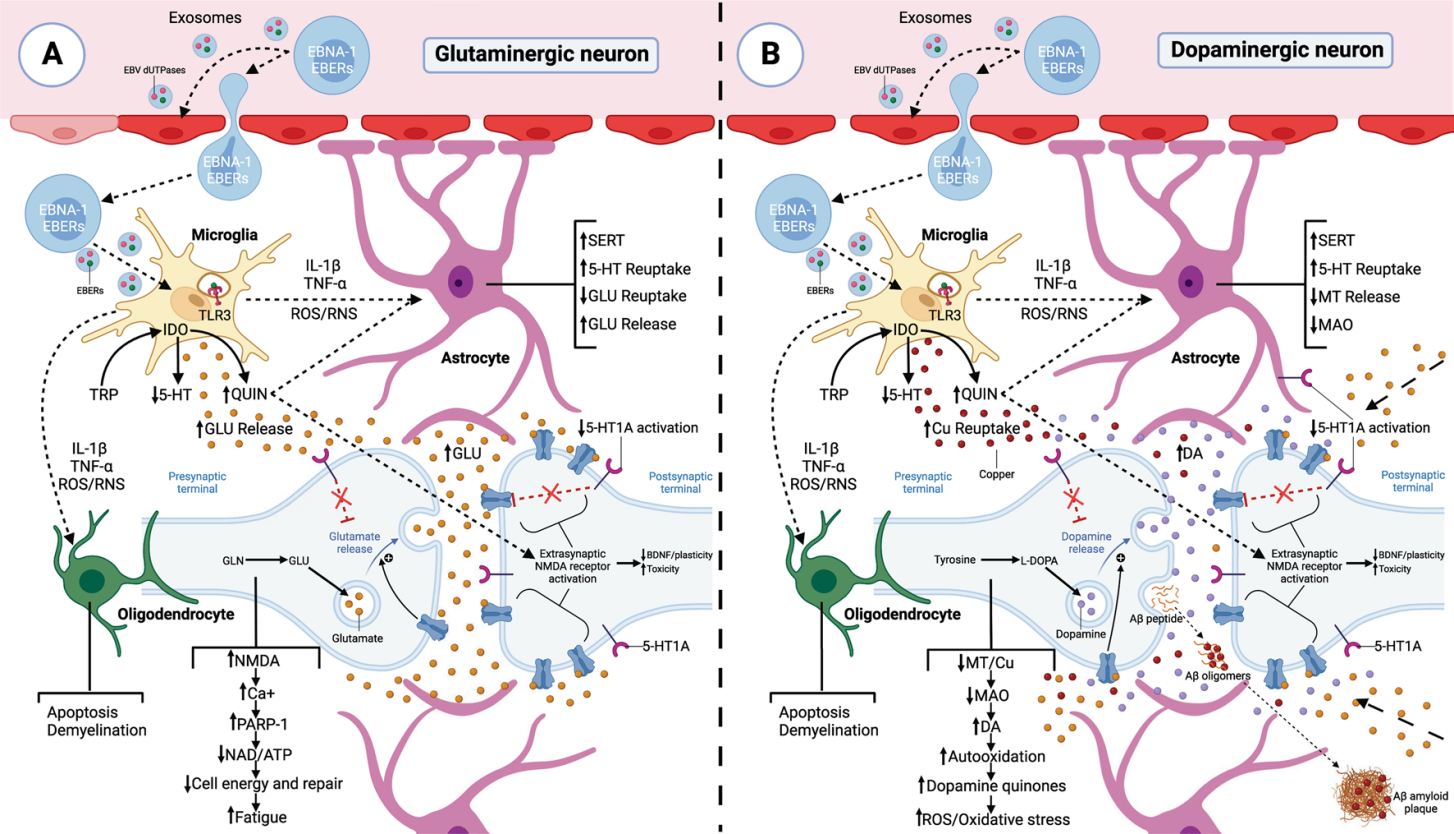
EBV infection and neuroinflammatory alterations: implications in neurotransmission and copper metabolism. **A** Exosomes with EBV dUTPases and EBERs from EBV-infected cells can reach the blood–brain barrier and contact endothelial cells causing activation of TLR2 receptors by dUTPases. The blood–brain barrier’s endothelial cells are activated and release proinflammatory cytokines that disrupt the blood–brain barrier’s integrity. Both EBV-infected cells crossing the blood–brain barrier and exosomes with viral genetic material can activate microglia through TLR3 receptors that detect the presence of EBERs. Activated microglia release proinflammatory cytokines IL-1β and TNF-α, which increase the expression and activity of the serotonin transporter SERT in astrocytes, causing an increase in serotonin (5-HT) reuptake and a decrease in its extracellular levels. Oligodendrocytes are particularly susceptible to inflammation. Overexposure to cytokines such as TNF-α can damage these cells, potentially leading to apoptosis and demyelination. Both increased proinflammatory cytokines and increased oxidative and nitrosative stress (ROS/RNS) from activation of microglia via TLR3 could lead to increased IDO activity in microglia, resulting in reduced tryptophan (TRP) levels, increased kynurenine catabolites and decreased 5-HT synthesis. Quinolinic acid (QUIN) stimulates glutamate (GLU) release by activating the NMDA glutamate receptor in the presynaptic neuron. On the other hand, in astrocytes it decreases the expression of glutamate transporters and increases their release, thus increasing extracellular glutamate levels. Thus, quinolinic acid can increase glutamate levels in the brain and decrease brain cells' ability to eliminate excess glutamate. Moreover, quinolinic acid has neurotoxic properties by binding to the neurons' NMDA receptor, followed by sustained calcium (Ca^2+^) influx leading to increased oxidative and nitrosative stress. This increase in nitrosative and oxidative stress leads to the activation of PARP-1 polymerase to prevent DNA damage. But continuous overactivation of PARP-1 leads to depletion of intracellular reserves of nicotinamide adenine dinucleotide (NAD) and ATP, with the consequent alteration in energy production and mitochondrial function. The binding of glutamate to extrasynaptic NMDA receptors can lead to a reduction in the levels of neurotrophic factors such as brain-derived neurotrophic factor (BDNF), leading to a decrease in synaptic plasticity and increased vulnerability to excitotoxicity. The decrease in 5-HT levels decreases the activation of 5-HT1A receptors allowing more glutamate to be released, as the activation of 5-HT1A receptors decreases the release of glutamate in presynaptic neurons. In addition, the activation of 5-HT1A receptors in the postsynaptic neuron inhibits the activation of NMDA receptors, so a decrease in extracellular 5-HT prevents the activation of 5-HT1A receptors allowing overexcitation of NMDA receptors by glutamate and quinolinic acid. **B** To the right, the same process occurs, but with alterations in dopaminergic neurons. The decrease in extracellular 5-HT levels due to increased 5-HT reuptake decreases the activation of 5-HT1A receptors in neurons allowing more dopamine (DA) to be released, as the activation of 5-HT1A receptors decreases the release of dopamine in presynaptic neurons. Additionally, the activation of 5-HT1A receptors in the postsynaptic neuron inhibits the activation of NMDA receptors, so a decrease in extracellular 5-HT prevents the activation of 5-HT1A receptors allowing overexcitation of NMDA receptors by quinolinic acid and excess glutamate released by glutaminergic neurons. In turn, the decrease in 5-HT levels decreases the activation of astrocytes’ 5-HT1A receptors, leading to less release of metallothioneins (MT) to the extracellular space. Metallothioneins are especially important to protect dopaminergic neurons from excess dopamine quinones. Consequently, a decrease in the availability of copper in these cells due to a decrease in metallothionein production and copper absorption in the intestine would lead to a decrease in dopamine breakdown via MAO, as this enzyme’s activity depends on copper. If MAO enzymes cannot efficiently break down dopamine, more dopamine may accumulate in the cytosol of neurons and undergo autoxidation. Then, in conditions of copper deficiency in dopaminergic neurons, there could be a greater propensity to form dopamine quinones and ROS due to the autoxidation of accumulated dopamine, causing mitochondrial dysfunction. In addition, the activation of microglia in response to inflammatory stimuli would increase copper uptake from the synaptic space by microglia and disrupt neuronal copper reuptake. This can decrease the amount of copper available for neurons, disrupting their ability to regulate NMDA receptor activity and leading to overactivation of NMDA receptors. Increased copper in microglia can reduce its ability to phagocytose unwanted proteins. This could lead to an accumulation of amyloid-beta (Aβ) protein released by neurons. These Aβ deposits also have a high affinity for copper, so a greater increase in Aβ in these areas would cause larger amounts of copper to deposit out of reach of neurons and even form Aβ amyloid plaques

Under normal conditions, 5-HT cannot cross the blood–brain barrier, so increases in its levels in peripheral blood would not increase CNS 5-HT levels [284]. Under conditions of chronic EBV infection, however, EBV dUTPase proteins could alter the integrity of the blood–brain barrier (Fig. 4), allowing the passage of proinflammatory cytokines, 5-HT, and EBV-infected cells [10]. If the CNS eventually becomes infected by EBV, TLR3 activation by EBERs released from latent cells would elicit a response opposite to that occurring in peripheral tissues [271, 285]. TLR3 activation in microglia in the CNS during brain infections (Fig. 1) results in increased release of IL-1β and TNF-α, which enhances SERT expression and activity in astrocytes and, therefore, increases reuptake and degradation of 5-HT to the metabolite 5-hydroxyindoleacetic acid (5-HIAA), thereby reducing extracellular levels of 5-HT [271, 285–288]. As a consequence, there would be a reduction in the activation of neuronal 5-HT1A receptors, possibly inducing the sensation of fatigue and the development of depression [285–287, 289]. This, added to the fact that the 5-HT synthesis rates in women are half that of men, could explain why women are more likely to suffer from depressive disorders during infectious processes [290]. In addition, activation of the 5-HT1A receptor is involved in ACTH secretion so that a decrease in 5-HT availability in the CNS would also translate into a decrease in 5-HT1A activation and lower ACTH synthesis [291]. Therefore, serotonergic alteration in the CNS also allows the maintenance of peripheral hypocortisolemia.

### Alteration of the microbiota

When there is chronic infection of the intestinal mucosa, the defense mechanisms involved in maintaining the intestinal barrier are compromised [292, 293]. On the one hand, serotonergic alteration caused by 5-HT accumulation in the intestinal mucosa generates chronic inflammation [271–274]. This intestinal inflammation (Fig. 1) causes a decrease in acid secretion from the stomach and in the expression of enzymes necessary to digest carbohydrates, causing more carbohydrates to reach the intestinal microbiota without being digested or absorbed [277, 294]. On the other hand, the increase in proinflammatory cytokines (IFN-γ and TNF-α) as a consequence of EBV infection of epithelial cells generates the rupture of tight junctions between enterocytes, leading to an increase in intestinal permeability [292]. Finally, impaired function of adaptive immunity prevents the resolution of EBV infection and an adequate response to other pathogens [171]. Therefore, increased nutrient influx for these commensal bacteria coupled with decreased acid secretion, barrier breakdown, and alteration of the intestinal adaptive immune system would lead to proliferation of these bacteria, resulting in the occurrence of small intestinal bacterial overgrowth (SIBO) and the development of food intolerances [294, 295]. In addition, as a consequence of the increased permeability, substances and microorganisms that should not cross the barrier would cross the barrier and therefore further activate innate immunity (TLRs), leading to greater accumulation of 5-HT and an increase in symptoms [293]. Another consequence of SIBO is bacterial deconjugation of bile salts, leading to impaired micelle formation, fat malabsorption, and deficiencies in fat-soluble vitamins (A, D, E, and K) [294]. Competitive uptake of vitamin B12 by bacteria results in less binding to intrinsic factor and therefore less absorption in the terminal ileum [294].

### Oxidative stress

When cells are infected by a virus, there is increased production of free radicals by the mitochondria, peroxisomes, endoplasmic reticulum stress, nicotinamide adenine dinucleotide phosphate hydrogen phosphate (NADPH) oxidase, metabolizing enzymes, and due to the Warburg effect (aerobic glycolysis), causing cells to become stressed and enter apoptosis [296–298]. However, viruses have evolved defense mechanisms that upregulate host cell protective systems against these free radicals [296, 299]. Normally, to avoid the harmful effects of ROS, cells upregulate their antioxidant defenses (both enzymatic and non-enzymatic) [296, 300]. During viral infections, however, the rate of intracellular ROS production exceeds the antioxidant capacity in cells, causing oxidative stress, which consequently leads to the depletion and deficiency of antioxidant enzymes, such as superoxide dismutase (SOD), catalase (CAT), and glutathione peroxidase (GPx), and the depletion of non-enzymatic free radical scavengers such as vitamin E (α-tocopherol), vitamin C (ascorbate), glutathione (GSH), β-carotene, and melatonin [296, 297, 299–301]. In the case of EBV, EBV-transformed cells have evolved resistance mechanisms against ROS and use the DNA damage caused by free radicals during oxidative stress to increase the expression of DNA methyltransferases, which induce epigenetic alterations by methylating viral DNA to decrease its replication and cause the virus to move into distinct latency phases (Fig. 1) that allow evading the immune system [299]. Specifically, EBNA-1 transcriptionally activates the catalytic subunit of NADPH oxidase NOX2, causing ROS accumulation and DNA damage to allow cell transformation [302]. One of the consequences of NADPH oxidase NOX2 activation is that increased ROS production can lead to insulin resistance by affecting insulin receptor signal transduction, causing a decrease in GLUT4 transporter expression and thus glucose uptake, thereby contributing to hyperinsulinemia [303, 304]. Therefore, during chronic EBV infection, the greater the number of infected cells, the greater the consumption of antioxidant substances and therefore deficiency.

### Transactivation of human endogenous retroviruses (HERV)

EBV can transactivate the env gene of HERV-K18 in infected B cells through the latent membrane proteins LMP-2A and LMP-1 [305]. HERV-K18 is a superantigen that causes stimulation of a large number of non-antigen-specific T cells through HLA-II and may facilitate persistent EBV infection and/or transmission, as B cell memory formation is CD4 T cell-dependent [305, 306]. For example, marmosets, New World primates, do not harbor HERV-K18 in their genome, and long-term persistent EBV infection cannot therefore be established, so this superantigen is thought to be involved in the establishment of EBV latency [306].

In addition, these long-term superantigens could also contribute to immune exhaustion and a state of unresponsiveness (anergy) on the part of T lymphocytes due to chronic exposure to these antigens [307].

### Microclot formation

The overproduction of proinflammatory cytokines TNFα, IL-6, and IL-1β during infectious processes causes hypercoagulation, platelet activation, leukocyte infiltration, and vascular hyperpermeability [231–233, 308]. When activated also through 5-HT2A receptors by the increase of 5-HT in peripheral blood or by the binding of EBERs to TLR3, platelets bind to fibrinogen, causing enhancing aggregation and coagulation processes [279, 309]. Upon activation, they can release β-amyloid (Aβ) peptide either directly or via cleavage of amyloid precursor protein [310]. A consequence of the increased production and release of Aβ in response to infection is that it interacts specifically with fibrinogen, causing fibrinogen oligomerization, fibrin(ogen) deposition, and Aβ fibrillation, ultimately favoring the formation of abnormal fibrin microcoagulates resistant to degradation by fibrinolytic enzymes [311]. The Aβ fibrinolytic pathway appears to be part of the innate immune response protecting against fungal, bacterial, and even viral infections such as herpesvirus infections, where oligomers bind to and entrap viral glycoproteins, accelerating β-amyloid deposition [312, 313].

In addition, increased levels of IL-1β, IL-6, and TNF-α stimulate the production of serum amyloid A (SAA) in hepatocytes [314]. The increase of SAA in blood may favor its interaction with fibrinogen, causing amyloidogenic changes in fibrinogen, leading to the formation of fibrin amyloid microaggregates resistant to fibrinolysis [78, 315].

In both cases, microclot formation leads to capillary obstruction (Fig. 1), which compromises blood flow and increases inflammation, contributing to the appearance of various symptoms [78, 147, 258].

Another factor influencing microclot formation is endothelial damage caused by EBV infection, either by positive upregulation of NOX2 by EBNA-1 in EBV-transformed endothelial cells or by activation of TLR3 by EBERs, where NOX2 activation causes vasoconstriction and thrombosis through platelet aggregation by increased overproduction of hydrogen peroxide, isoprostane, or inactivation of nitric oxide [256, 302, 316, 317].

### IDO activation

During persistent CNS infections (Fig. 4), aside from increased 5-HT reuptake in astrocytes, increased oxidative and nitrosative stress leads to activation of indoleamine 2,3-dioxygenase (IDO), and increased proinflammatory cytokines activate tryptophan 2,3-dioxygenase (TDO) [318]. Both IDO and TDO activation consume and therefore decrease the levels of tryptophan and lead to increased tryptophan catabolite (Fig. 1) synthesis, further contributing to the extracellular decrease in 5-HT levels [318]. As melatonergic pathways depend on the availability levels of 5-HT as a necessary precursor, a decrease in 5-HT would lead to a reduction in melatonin production [318]. Thus, a reduction in melatonin levels contribute to the development of sleep disorders and depression [318]. In addition, reduced levels of melatonin could help to maintain hyperinsulinemia during the night in chronic infections, since insulin secretion is inhibited by its binding to the melatonin receptor on pancreatic β-cells [319].

Both elevated IFN-γ, TNF-α, and IL-6 levels and increased ROS in EBV-infected cells leads to increased IDO activity and thus reduced levels of tryptophan as a consequence of its increased catabolism [202, 320]. Reduced glucose uptake due to insulin resistance and reduced tryptophan levels is a defense mechanism used by infected cells to increase cellular stress and suppress viral replication [320]. For example, induction of IDO activity by IFN-γ and TNF-α has been observed to be responsible for inhibition of herpes simplex virus replication [321]. However, chronic viral infections take advantage of the immunosuppressive effect of kynurenine catabolites produced from tryptophan catabolism to create a state of disease tolerance [320]. The overexpression of IDO, both in uninfected antigen-presenting cells—induced by IFN-γ, TNF-α, and IL-6 or by activation of RIG-I/TLR3 by EBERs or by activation of TLR2 by EBV dUTPase—as well as in EBV-transformed antigen-presenting cells—induced by TNF-α and IL-6 via MAPK/p38 and NF-κB—suppresses the activation and proliferation of antiviral T cells (CD4 and CD8) and decreases the cytotoxic activity of CD8 T cells by binding kynurenine catabolites released by antigen-presenting cells to the aryl hydrocarbon receptor (AhR) [10, 202, 320, 322, 323]. Increased levels of kynurenine catabolites together with tryptophan depletion in the local microenvironment result in metabolic stress on CD4 T cells, leading to impaired T-cell metabolism, impaired antigen-specific response, cell cycle arrest and apoptosis of Th1 cells, inhibition of Th17 cell differentiation, and promotion of Th2 cell polarization [320, 323–325]. In addition, upon binding to AhR, kynurenine metabolites also increase T-cell differentiation into FoxP3+ regulatory T cells versus Th17 cells, causing an increase in their expansion and IL-10 release, which suppresses Th1 responses [320, 323, 324]. In the case of EBV-transformed B cells, IDO activation, through the release of IL-10 and TGF-β, suppression of Th1 and Th17 cells, and conversion of CD4+ T cells to Treg, can trigger the same effects on CD4 T cells as elicited by other antigen-presenting cells [325]. Even kynurenine produced by these EBV-transformed B cells inhibits NKG2D expression in nearby NK cells through the c-Jun N-terminal kinase pathway to escape NKG2D-mediated attack by immune cells [320, 326].

In addition, the cytokine microenvironment produced by antigen-presenting cells for Th1/Th2 differentiation of Th0 cells is dependent on the oxidative state of the cell, where the depletion of antioxidant substances such as glutathione inhibits the production of Th1-associated cytokines and favors the production of Th2-associated cytokines [327]. Therefore, the continuous increase of free radicals in EBV-transformed antigen-presenting cells leads to the depletion of antioxidant substances, resulting in increased Th2 differentiation.

In summary, through tryptophan degradation resulting from increased IDO and ROS activity in antigen-presenting cells, EBV-infected ectopic lymphoid aggregates can enhance differentiation into Th2 over Th1 and into Treg over Th17, thus creating an environment dominated by the release of anti-inflammatory cytokines (IL-10, IL-4, and TGF-β) that decreases the antiviral adaptive response and favors immune tolerance and the development of autoimmune diseases [325], as it could lead to an increase in EBV-infected cells that could present autoantigens or viral EBNA-1 through MHC-II, which can undergo posttranslational modifications and form neoantigens that generate autoreactive responses [171, 212–215]. All this contributes to the withdrawal of immune surveillance of EBV-transformed cells and to the chronification of infection.

### Hypozincemia

Another consequence of elevated levels of proinflammatory cytokines such as IL-1 and IL-6 is the appearance of hypozincemia due to increased expression of zinc transporters and zinc-binding peptides (metallothioneins and alpha2-macroglobulin), which sequester and redistribute zinc to tissues (Fig. 1), thus decreasing its availability in blood [328, 329]. Hypozincemia is usually transient and resolves when the inflammatory response disappears, releasing zinc from the cells into the serum [328]. Transient hypozincemia is a defense mechanism to decrease the availability of zinc (Zn) to extracellular microorganisms and increase available Zn in cells of the immune system [328]. Within these immune cells, it allows intoxication of engulfed pathogens on the one hand and combating oxidative/nitrosative stress to prevent cell DNA damage on the other hand [328]. However, some viruses can use the increased intracellular Zn to their advantage and generate chronic hypozincemia [330]. Because intracellular free Zn is toxic to the cell, under normal conditions, it is mostly bound to proteins such as metallothioneins (MTs), whose expression is induced by increased intracellular Zn [330]. MTs are cysteine-rich proteins that regulate Zn and copper homeostasis, are involved in heavy metal detoxification (such as cadmium and mercury), alleviate oxidative/nitrosative stress, and participate in immune responses [331]. Viruses depend on intracellular zinc, as it is utilized by newly synthesized viral proteins [330]. Consequently, cellular systems that control Zn homeostasis, such as MTs, could constitute a protective barrier against viral replication [330]. In the case of chronic EBV infection, increases in proinflammatory cytokines IL-1, IL-6, and IFN-γ, such as from EBERs, may induce increased expression of zinc transporters and metallothionein production, especially in the liver [236, 329]. In EBV-transformed cells, LMP1 mimics the CD40–CD40L interaction, activating the nuclear enhancer factor kappa light chain enhancer of activated B cells (NF-κB) [332]. Activation of NF-κB induces several antiapoptotic proteins, stimulates cell cycle progression, and plays a crucial role in carcinogenesis [332]. Increased levels of free zinc could serve as a co-stimulus for the upregulation of NF-κB produced by LMP1 or CD40 signaling, as the intracellular increase in zinc induces the expression of MTs, which elevates the DNA-binding activity of NF-kB [332, 333]. Thus, EBV-infected cells require zinc for increased overexpression of MTs and for transactivation of EBNA-1, thereby increasing their intracellular levels by increasing the expression of zinc transporters and favoring zinc release from metallothioneins via the action of increased ROS, ultimately aiding in the activation, proliferation, and immortalization of transformed B cells [330, 332–334]. Therefore, in the long term, overexpression of MTs and zinc transporters reduces the availability of zinc in blood and its uptake by immune cells, leading to altered immune responses and downregulation of intracellular antioxidant activity [236]. Increased overexpression of MTs in cases of serum zinc deficiency causes hypoplasia of primary and secondary lymphoid organs (thymus, spleen, lymph nodes, and Payer’s plaques), decreased secretion of Zn-thymulin in the thymus, a decrease in the number of total T lymphocytes, altered T-helper activation and function, reduced cytotoxic activity of NK cells, Th1/Th2 imbalance with increased release of Th2 cytokines, and polarization of Treg versus Th17 cells, ultimately leading to a state of immunosuppression with low resistance to infections [236, 329, 335]. In addition, both the elevated levels of extracellular MTs that attract leukocytes to the site of inflammation [336] and the increased expression of MHC-II in cells due to intracellular zinc deficiency as a consequence of chronic hyponzinemia [329] allow infection of a greater number of cells by EBV.

### Ceruloplasmin deficiency

**Fig. 5:**
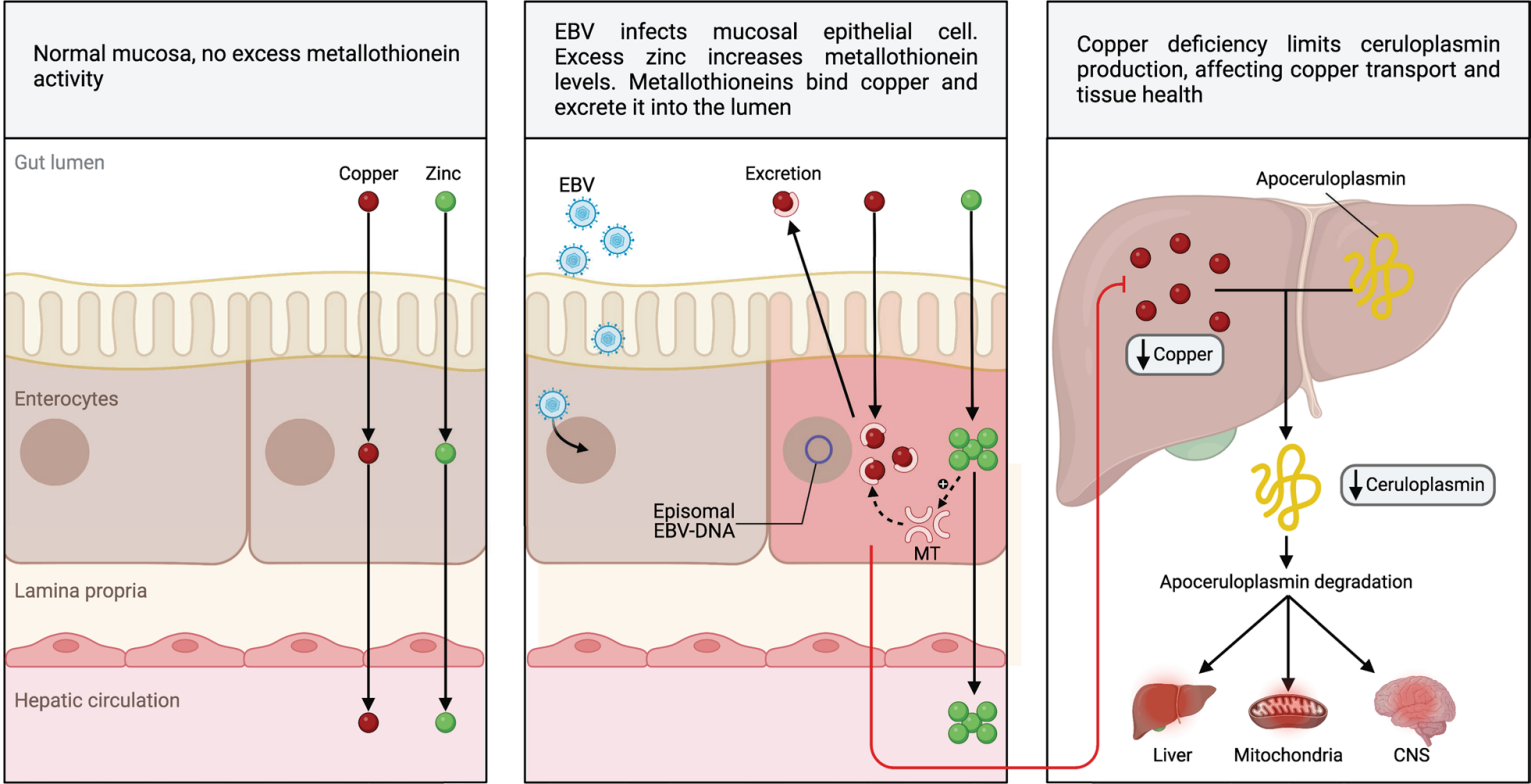
EBV infection generates copper dysregulation in tissues. EBV infection increases zinc uptake, stimulating metallothionein expression, which displaces copper and affects ceruloplasmin synthesis and copper distribution in tissues

MTs can also bind copper (Cu) [337]. Inflammatory processes caused by an infection in the intestinal mucosa could lead to overexpression of intracellular MTs in enterocytes (Fig. 5) as a response to increased intracellular Zn to counteract the damaging effects of increased free radicals, causing these MTs to also bind Cu from food, since MTs have a higher affinity for Cu than for Zn [337–339]. Thus, the increase in intracellular Zn would cause Cu to be retained in enterocytes, preventing its absorption and leading to Cu deficiency in the body (Fig. 1) [337, 339]. The involvement of Zn in Cu absorption can be observed in patients with Wilson’s disease. These patients are treated with large doses of Zn to increase the expression of MTs and reduce Cu absorption in the intestinal mucosa, as these patients accumulate excessive amounts of Cu due to their low levels of ceruloplasmin [337].

Decreasing Cu absorption could result in decreased synthesis of ceruloplasmin [340–342], a protein that allows Cu transport into tissues and is overexpressed during infections and inflammatory processes in response to increased levels of IL-6 and IFN-γ [342, 343]. Ceruloplasmin deficiency could generate alterations induced by oxidative stress by decreasing the transport of Cu needed as a cofactor for some enzymes, such as superoxide dismutase (CU/Zn-SOD) and cytochrome C oxidase [344].

One of the organs that may be most affected by this deficiency is the brain, as this organ has a high respiratory rate and is prone to oxidative stress [344]. Therefore, a decrease in Cu transport in the brain could lead to an increase in oxidative stress as a consequence of increased release of ions that contribute to the formation of free radicals, a decrease in endogenous antioxidant systems (CU/Zn-SOD), and an increase in iron accumulation by decreasing the ferroxidase activity of ceruloplasmin [344]. This increased oxidative stress due to ceruloplasmin deficiency could lead to a loss of acquired language and memory skills, as occurs in Parkinson's and Alzheimer’s diseases [344, 345]. In the hippocampus, neurons use copper to attenuate *N*-methyl-d-aspartate (NMDA) receptor-induced excitotoxicity by decreasing cytoplasmic Ca^2+^ concentration after NMDA receptor activation [346]. Therefore, if microglia are activated in response to inflammatory stimuli (via TLR3 in the presence of viral genetic material) in brain areas where copper is used to prevent NMDA receptor excitotoxicity (Fig. 4), it would increase Cu uptake from the synaptic space by microglia and disrupt Cu reuptake by neurons [346]. This would generate an overactivation of NMDA receptors and could subsequently lead to neurodegeneration [346]. In addition, increased intracellular Cu reduces the phagocytic properties of microglia, which may lead to increased Aβ deposition. These Aβ deposits also have high affinity for Cu, so a further increase in Aβ in these areas would cause greater amounts of Cu to be deposited out of reach of neurons [346].

Substantial oxidative stress also occurs in the intestine due to exposure to foreign substances and microbial pathogens [347]. Therefore, a decrease in the antioxidant system could lead to increased damage caused by oxidative stress, resulting in inflammation, intestinal barrier dysfunction, and immune imbalance [347]. In addition, a decrease in copper transport could also lead to a decrease in diamine oxidase (DAO) activity [348, 349], altering histamine metabolism and generating the appearance of symptoms caused by its accumulation [350]. On the other hand, copper also contributes to the activity of monoamine oxidases (MAOs), which are involved in the oxidative deamination of mitochondrial monoaminergic neurotransmitters, such as dopamine, serotonin, tyramine, and norepinephrine [351]. Therefore, copper transport deficiency could result in decreased MAO activity in enterocytes, contributing to the accumulation of 5-HT in the intestinal mucosa that is also generated by the reduction in SERT activity [352].

At the immunological level, Cu deficiency may generate a deficiency in CD4 T-cell responses to mitogens [353], immune hyporesponsiveness (immune tolerance) [354], and an increased humoral response [355], again supporting the presence of an immunodeficiency. Therefore, overexpression of MTs with serum zinc deficiency together with ceruloplasmin deficiency could lead to a deficiency in T-lymphocyte response.

In women, this ceruloplasmin deficiency may be aggravated during menopause, where decreased estrogen causes decreased ceruloplasmin synthesis [356].

## Role of EBV in ME/CFS and long COVID

Among the immunological alterations presented by ME/CFS patients, there is reduced cytotoxic response by T lymphocytes and natural killer cells (NKs) [1, 35, 44, 357], reduced T-cell responses to mitogens and other specific antigens with decreased activation [1, 35, 358], deficient EBV-specific B- and T-cell response [21], increased Th2 response (especially IL-10) [35, 36, 38, 41, 46, 359], increased regulatory T cells (CD4+CD25+FoxP3+) [359], and increased IFN-γ levels [359]. In individuals with ME/CFS, ancestral HLA-II haplotypes, and latent EBV, these immunological alterations can be explained by the EBV-acquired immunodeficiency model, where the impairment of CD4 T-cell function and activation caused by these ancestral HLA-II haplotypes could generate T-cell depletion and thus an increase in EBV-infected ectopic lymphoid structures [8, 171]. These infected tissues, when not controlled by the adaptive response, could end up with chronic inflammation due to the activation of TLR3 receptors or RIG-I, which results in continuous detection of viral genetic material (EBERs) [231–233], causing a chronic innate response and inflammation through the continuous release of proinflammatory cytokines, generating an increase in IL-1, IL-6, TNFα and IFN-γ in individuals with ME/CFS [37]. Chronic release of proinflammatory cytokines of innate immunity, increased Treg cell activity, aerobic glycolysis of infected cells, and activation of neutrophils by ANCA lead to excessive ROS production, and this could explain the increased oxidative and nitrosative stress, depletion of antioxidant substances, increased aerobic glycolysis in circulating cells, and increased damage to fatty acids, DNA, and proteins that occur in patients with ME/CFS [296–298, 360]. This increased oxidative and nitrosative stress could be exploited by EBV-infected cells for transformation by methylation of viral DNA, preventing its replication and favoring the establishment of different types of latency that allow evading the immune system [299]. Furthermore, increased IL-10 levels in ME/CFS patients support the presence of a persistent infectious state and decreased activity of T cells and NKs [359].

Increased levels of TNF-α and IFN-γ in ME/CFS also explain the increased intestinal inflammation and permeability, development of irritable bowel, and increased bacterial translocation in these patients [35, 292, 359].

This acquired immunodeficiency could also justify the lytic or abortive reactivation of this and other latent pathogens, such as other herpesviruses and Parvovirus B19, that occurs in patients with ME/CFS [9, 10, 15, 17, 34, 361–363] and could generate, on the one hand, the production of transient autoantibodies such as ANCAs, ANAs, or antiphospholipid antibodies (such as anticardiolipin) [71, 360], causing exacerbation of symptoms, and on the other hand, the increased release of viral dUTPases that increase neurological symptoms [10, 11, 26], generating more inflammation and disease flares.

Patients with ME/CFS may present with insulin resistance, increased blood insulin levels, high waist circumference, high triglycerides, and hypocortisolism [47–60, 364]. Chronic elevated levels of insulin and proinflammatory cytokines could explain the negative regulation of cortisol secretion, where increased insulin causes a deficient cortisol response to hypoglycemia, as occurs in patients with ME/CFS assessed using the insulin-induced hypoglycemia test [54, 58, 253, 262, 263, 365]. Thus, it appears that patients with ME/CFS attempt to maintain a state of transient hypoglycemia caused by increased proinflammatory cytokines, hyperinsulinemia, and hypocortisolism to maintain a chronic innate immune response, decreased viral replication, and enhanced CD8 T-cell effector response [247–249], though without being able to resolve the infection by presenting an alteration in CD4 T cell function and, therefore, a deficient cytotoxic response of CD8 T cells in individuals with “weak” HLA-II haplotypes against EBV. The increase of EBV latent cells in different tissues could lead to a more severe state of insulin resistance and, thus, to an increased risk of developing metabolic syndrome, type 2 diabetes mellitus, cardiovascular disease and Alzheimer’s disease in these patients [58].

Within the serotonergic alterations presented by patients with ME/CFS, it has been suggested that viral infection may cause damage to the blood–brain barrier, leading to the entry of viral genetic material that activates TLR3 receptors in microglia, leading to increased release of IL-1β and TNF-α, which increases SERT expression and activity in astrocytes and, therefore, increases 5-HT reuptake and its degradation to the metabolite 5-HIAA, thereby reducing extracellular levels of 5-HT [271, 285–288]. This would result in reduced activation of neuronal 5-HT1A receptors, possibly inducing the sensation of fatigue and the development of depression [285–287, 289]. Furthermore, during infectious processes in ME/CFS patients, both increased proinflammatory cytokines and increased oxidative and nitrosative stress due to activation of microglia via TLR3 could lead to increased IDO activity in microglia, resulting in reduced tryptophan levels (increased its catabolism), increased kynurenine catabolites, and decreased 5-HT and melatonin synthesis [366, 367]. Depletion of tryptophan together with increased kynurenine catabolites could lead to an impaired Th1 cell response and increased promotion of Th2 cells, favoring immune tolerance to the disease [325, 366]. Furthermore, within kynurenine metabolites, quinolinic acid has been shown to be an NAD+ precursor that has neurotoxic properties through its binding to the *N*-methyl-d-aspartate (NMDA) receptor, which leads to sustained Ca^2+^ influx and thus increased nitrosative stress [366]. This increased nitrosative and oxidative stress results in activation of poly(ADP-ribose) polymerase-1 (PARP-1) to prevent DNA damage [366]. Continued overactivation of PARP-1, however, results in depletion of intracellular NAD+ and ATP stores with consequent impairment in energy production and mitochondrial function, which have been reported in ME/CFS patients [366]. In addition, quinolinic acid stimulates synaptosomal glutamate release by acting as an NMDA receptor agonist and, in astrocytes, decreases the expression of glutamate transporters and increases their release, thereby increasing extracellular glutamate levels [367, 368]. Elevated glutamate, apart from generating neurotoxicity, can cause mitochondrial dysfunction and lower energy [366]. Therefore, increases in kynurenine and its metabolites resulting from increased IDO activity could contribute to neuroinflammation, increased glutamate, altered immune system, depression, altered gut microbiota, and fatigue in ME/CFS patients [366, 367, 369]. However, it is not only increased quinolinic acid that can explain the overstimulation of NMDA receptors. In brain areas of increased Cu use, ceruloplasmin deficiency and activation of microglia in response to the presence of viral genetic material could also contribute to the overstimulation of NMDA receptors, increased levels of nitric oxide/peroxynitrite levels, and the cognitive impairment present in ME/CFS patients [346, 369, 370].

Decreased activity of Cu-dependent enzymes, such as SOD, and decreased levels of ceruloplasmin in patients with ME/CFS [371] can be explained by a deficiency in Cu uptake due to overexpression of MTs in enterocytes in response to mucosal infection. This Cu deficiency could cause a decrease in ceruloplasmin synthesis and a decrease in the activity of Cu-dependent enzymes, contributing to increased oxidative stress.

On the other hand, the hypozincemia observed in patients with ME/CFS could occur due to increased proinflammatory cytokines in response to infection, as they increase the expression of zinc transporters and zinc-binding peptides, leading in the long term to a reduction in their availability in blood and their uptake by immune cells [328, 329, 372]. This results in a decrease in their activation in the presence of antigens and altered immune responses with downregulation of intracellular antioxidant activity [236, 329, 335, 372]. In addition, this deficiency could lead to increased MHC-II expression allowing infection of a greater number of cells by EBV in these patients [329].

Another abnormality present in patients with ME/CFS is the presence of fibrinolysis-resistant amyloid fibrin microbleeds and platelet hyperactivation [78], which could also be a consequence of the overproduction of proinflammatory cytokines or due to the binding of EBERs to TLR3 [231–233, 308, 309].

In the case of LC, there is increased levels of depleted T cells (PD-1+/Tim-3+) [128], impaired T-cell function [119, 128], decreased recruitment of activated T cells [125], impaired cytotoxic response of NK cells [373], increased Th2 response [128], increased Treg cells [374–376], increased proinflammatory cytokines (IFN-γ, TNFα, IL-1, and IL-6) and IL-10 [118, 125, 377, 378], increased oxidative stress [377, 379–381], decreased antioxidant defenses [380], hypozincemia [380, 382], hypocortisolism [128, 133, 141–143], presence of amyloid fibrin microbleeds [33, 146–149], presence of transient autoantibodies (ANAs, ANCA and anti-cardiolipin) [106, 113, 383, 384], insulin resistance [385, 386], and increased IDO activity and kynurenine levels [387].

Just as the binding affinity of EBV peptides to MHC class II molecules can influence the generation of an antiviral response [191, 218], it could also occur with SARS-CoV-2 [388]. It is known that single amino acid alterations in immunogenic peptides can enhance or decrease the binding of the peptide to the MHC class II molecule, causing the differentiation of CD4 T cells into Th1 cells if the binding is strong or into Th2 cells if the binding is weak [389]. Therefore, having weak HLA-II haplotypes against SARS-CoV-2 could influence the loss of binding affinity between MHC class II molecules and viral peptides, leading to decreased immune protection and viral recognition [388]. For example, mutations in the Spike protein in Omicron cause the HLA-DRB1*03:01 allele to lose affinity for this protein [390]. Therefore, individuals with LC could have “weak” HLA-II haplotypes against SARS-CoV-2 different from those against EBV or they could be the same weak haplotypes against EBV. More research is needed in this area. In both cases, there would be impaired control of SARS-CoV-2-infected cells by CD4 T cells, as these are essential for their immunosurveillance and for maintaining CD8 T cell cytotoxic responses [230, 391]. Therefore, chronic antigenic stimulation by SARS-CoV-2 in the absence of CD4 T-cell support results in a loss of immunosurveillance of infected cells, generating chronic SARS-CoV-2 infection with increased viral reservoirs in tissues and leading to immune “exhaustion” by chronic exposure to these viral antigens, as occurs in other chronic infections [227, 230]. This immune exhaustion would lead to a decrease in the formation of memory T cells and an increase in the generation of short-lived effector cells and exhausted T cells, where these exhausted antiviral T cells, unable to become activated, are functionally ineffective and unable to exert their cytotoxic functions in response to antigenic stimulation [227, 229], resulting in impaired immunosurveillance of SARS-CoV-2-infected cells and other cells with latency to another virus, such as EBV [128, 139]. This could result in the recruitment of EBV-latent leukocytes to inflamed tissues, leading to the formation of EBV-infected ectopic lymphoid structures and the development of EBV-acquired immunodeficiency as in post-EBV-infected ME/CFS. This cellular impairment of EBV control can be observed by increased antibody responses to EBV and increased reactivation in patients with LC [8, 128–130, 133–140]. It has also been observed that symptoms of fatigue and neurocognitive dysfunction in patients with LC are associated with EBV reactivation [138].

Another commonality of both diseases is that women are at higher risk of developing them than men [35, 392]. This may be because estrogens decrease the CD4/CD8 T lymphocyte ratio, increase B-cell survival, antibody release, and HLA-II expression, and decrease the Th1 antiviral response [209–211]. In addition, TLR3 expression on platelets is higher in women, thus increasing the risk of increased platelet hyperactivation and hypercoagulability [279].

In summary, according to the model presented here, ME/CFS develops in three steps: first, an acquired EBV immunodeficiency develops in individuals with “weak” HLA-II haplotypes against EBV, which prevents control of latency I cells. Second, ectopic EBV-latent lymphoid structures form in different tissues (including the CNS) that promote inflammatory responses and further impairment of cell-mediated immunity. Third, immune exhaustion occurs due to chronic exposure to viral antigens and the disease consolidates. In the case of LC, before the first step there is prior infection with SARS-CoV-2 in individuals with “weak” HLA-II haplotypes against this virus and/or EBV.

## Treatments

Based on this model of development of both diseases, one might think that treatment with hydrocortisone would be beneficial to resolve hypocortisolism, but it has been observed to have limited and even adverse effects in patients with ME/CFS, since hypofunction of the HPA axis is the consequence, and not the cause, of immunological alteration [54]. Moreover, given the increased Th2 response and impaired Th1 antiviral response, the use of hydrocortisone could further potentiate this immunodeficiency and EBV reactivation [393, 394].

Given that there could be an increase in 5-HT levels in inflamed tissues and peripheral blood but a decrease in levels in the CNS, it could be thought that the use of selective serotonin reuptake inhibitors (SSRIs) could help to restore serotonergic impairment. At the CNS level, long-term use of SSRIs could increase extracellular levels of 5-HT by inhibiting SERT, preventing it from degrading to 5-HIAA and thus making more 5-HT available to bind to neuronal 5-HT1A receptors and improve fatigue [395]. At the peripheral level, however, it could be detrimental because elevated peripheral blood 5-HT levels could be involved in increasing insulin and 5-HT release by pancreatic β-cells via the 5-HT3 receptor and inhibition of glucagon secretion by α-cells, resulting in exacerbation of hyperinsulinemia and hypoglycemia [282, 283]. This, together with the fact that SSRIs also cause increased ROS and oxidative damage in β-cells, could result in an increased risk of developing type 2 diabetes in the long term [396]. This could occur for example with fluvoxamine treatment. Khani and Entezari-Maleki suggested that fluvoxamine, an S1R agonist, could alleviate the symptoms of LC associated with EBV reactivation [131, 132]. It would do so by reducing the activation of XBP1 and the endoplasmic reticulum stress response [131, 132]. As an SSRI, it could also enhance the availability of 5-HT in the central nervous system [397]. However, it might exacerbate intestinal symptoms and inflammation due to an increase in the availability of 5-HT at the intestinal level. Therefore, this aspect should be taken into account when considering the use of fluvoxamine in this context.

Given the increased insulin resistance due to infection and increased blood insulin, treatment with metformin could be beneficial, as it decreases lipogenesis and gluconeogenesis, improves insulin sensitivity, inhibits complex I of the respiratory chain, reduces ROS production, improves antioxidant activity, and suppresses the production of proinflammatory cytokines by macrophages [398, 399]. Of particular interest is that it is able to downregulate NADPH oxidase [399], thus preventing the transformation of EBV-infected cells by inhibiting the intracellular ROS production and DNA damage necessary for generating latency. It would also reduce microclot formation due to endothelial damage caused by the positive regulation of NOX2 by EBNA-1 in EBV-transformed endothelial cells or by the activation of TLR3 by EBERs [256, 302, 316, 317]. Furthermore, metformin does not stimulate endogenous insulin production, so it is not associated with a risk of hypoglycemia [399]. The beneficial effect of metformin has been observed, for example, in patients with fibromyalgia, in cultured skeletal muscle cells from patients with ME/CFS, and even in reducing the severity of acute SARS-CoV-2 infection [400–402].

The use of antioxidant supplements such as N-acetylcysteine (NAC), could also help to decrease oxidative stress, as they prevent the proapoptotic effect due to oxidative and nitrosative damage generated by increased tryptophan catabolites or increased NOX2 activity [327]. Another advantage of the use of antioxidants is the reduced risk of developing EBV-associated cancer [301]. NAC can inhibit NF-κB activation, thereby decreasing the resistance and survival of EBV latency cells [403, 404]. In addition, NAC suppresses the induction of Th17 cells and Tregs and promotes Th1 responses [327]. It even reduces glutamate levels in the CNS, thus reducing its neurotoxicity [405]. It has also been shown to be effective in reducing chronic inflammation and leukocyte recruitment to inflamed tissue caused by persistent EBV infection [404]. Other antioxidant supplements such as ubiquinol, Zn, vitamin E, vitamin C, and melatonin would also increase antioxidant defenses, preventing their depletion and reduce chronic oxidative stress due to infection [296, 297, 299–301]. Specifically, Zn could also suppress the expression of proinflammatory cytokines and MHC-II on the surface of antigen-presenting cells, preventing them from being infected by EBV [329]. It would even increase blood zinc values in individuals with hypozincemia, restoring its uptake by immune cells and improving their activity [236]. It has been observed that zinc supplementation improves glucose metabolism and insulin sensitivity in patients with diabetes and could thereby also prevent its development [406].

This observation suggests that the use of zinc ionophores such as hydroxychloroquine could be useful, since they increase intracellular Zn, decrease the expression of MHC-II, and inhibit the replication of SARS-CoV-2. On the other hand, they promote the reactivation of EBV and other herpesviruses, which could contribute in the long term to the development of lymphomas [407, 408]. Another benefit of hydroxychloroquine is that it inhibits TLR3 activation and therefore also reduces the inflammatory response and development of coagulation disorders [308]. Among its major disadvantages is that it limits antigen-specific T-cell responses, reduces Th1 responses, and inhibits antioxidant production, thereby increasing oxidative stress [409, 410]. Therefore, the disadvantages of hydroxychloroquine use outweigh its advantages, preventing its use for relieving EBV-acquired immunodeficiency and potentially worsening the condition of patients with LC or ME/CFS [410].

Treatment with antivirals such as valganciclovir or valacyclovir could reduce lytic and abortive reactivation, inflammation, the appearance of transient autoantibodies, disease flares, and insulin resistance [244, 247].

There are contradictions in the use of hyperbaric oxygen therapy, since on the one hand the increased oxidative stress it generates allows for increased pathogen clearance, synthesis of various growth factors, and angiogenesis [411]. On the other hand, increased oxidative stress can generate higher levels of ROS and reactive nitrogen species, leading to increased oxidative and nitrosative damage, mitochondrial aging, DNA damage, and maintenance of chronic inflammation [411]. The benefit of this therapy may lie in the fact that generated hyperoxia stimulates the release of IκBα, which inhibits and reduces levels of NF-κB, thereby resulting in lower transcription rates of genes for proinflammatory cytokines (IFN-γ, TNF-α, IL-1β, IL-6, and IL-8), ultimately decreasing inflammation [411]. In addition, the lack of oxygen transport due to vascular damage and microclot obstruction that these patients present could be resolved by improving tissue oxygenation through a combination of hyperoxia and hyperbaric pressure [412, 413]. This could also prove beneficial by decreasing replication in those tissues with lower oxygen tension, such as the brain and some mucous membranes (oral and digestive), where conditions of relative hypoxia allow certain viruses, such as EBV, to enter the lytic phase and replicate and thus generate reservoirs and greater inflammation in these tissues [414–416]. Therefore, this therapy could be useful for those viruses that do not generate latency, such as SARS-CoV-2, but could be detrimental for viruses that do, since it promotes the increase of latent cells by increasing oxidative stress.

Therefore, the symptoms of individuals with EBV-acquired immunodeficiency could be improved through the combined chronic use of antioxidant supplements, antivirals, and metformin, at least until a treatment is found that completely eliminates EBV latency cells. The use of anticoagulants to reduce thrombotic sequelae and improve the quality of life of patients with LC and ME/CFS could also be considered [78, 417].

## Summary

EBV infection in individuals with “weak” EBV HLA-II haplotypes prevents the control of latency I cells (Fig. 1). The escape of these cells from immunosurveillance results in the formation of ectopic EBV-latent lymphoid structures in different tissues that promote inflammatory responses by releasing EBERs, producing abortive reactivation, and releasing new virions. Increased viral reactivation may increase the production of transient autoantibodies and the release of viral dUTPases. The increase in EBV latent cells also leads to an increase in evasion mechanisms by decreasing the activation and cytotoxic response of EBNA-1-specific CD4 T cells through IL-10 release, Treg recruitment, and the release of EBV miRNA contained in exosomes. Activation of TLR3 by EBERs or of TLR2 by EBV dUTPases results in the release of proinflammatory cytokines (IL-1β, IL-6, IL-8, IL-12, TNF-α, and IFN-γ) and IL-10. Hypozincemia could occur due to increased proinflammatory cytokines, as they increase metallothionein expression, causing a reduction in their availability in blood and their uptake by immune cells. The increase of metallothioneins in enterocytes reduces Cu uptake and ceruloplasmin synthesis. Decreased Cu transport results in decreased activity of Cu-dependent enzymes (CU/Zn-SOD, DAO, MAO, and cytochrome C oxidase). In addition, increases in proinflammatory cytokines leads to the formation of amyloid fibrin microclots resistant to fibrinolysis, either by increased production of serum amyloid A (SAA) or by the release of β-amyloid (Aβ) peptide by platelets, causing capillary blockage. High levels of IFN-γ produced by NK cells in response to persistent infection cause insulin resistance by downregulating insulin receptor transcription in myocytes. To compensate for insulin resistance, the pancreas increases insulin production, resulting in compensatory hyperinsulinemia. Hyperinsulinemia reduces glycogenolysis in the liver, leading to transient hypoglycemia and decreased metabolism in peripheral tissues, resulting in exercise intolerance. Hyperinsulinemia can decrease ACTH secretion and, together with chronic exposure to IL-10, TGF-β1 and TNFα could also decrease cortisol secretion. Activation of TRL2 and TLR3 also causes inhibition and decreased expression of intestinal SERTs, resulting in extracellular accumulation of 5-HT and activation of 5-HT receptors, generating increased motility, nausea, and intestinal secretions. Intestinal serotonergic alteration together with the increase in proinflammatory cytokines also generates chronic inflammation, resulting in decreased acid secretion, breakdown of the intestinal barrier, decreased expression of enzymes necessary to digest carbohydrates, and alteration of the intestinal adaptive immune system, leading to the appearance of food intolerances and bacterial overgrowth in the small intestine. The occurrence of SIBO results in malabsorption of fat-soluble vitamins and vitamin B12. When activated via 5-HT2A receptors by increased 5-HT in peripheral blood or by binding of EBERs to TLR3, platelets bind fibrinogen, leading to increased aggregation and coagulation processes. Activation of TLR3 receptors in microglia causes increased release of IL-1β and TNF-α, which increases SERT expression in astrocytes and, therefore, 5-HT reuptake and its degradation to the metabolite 5-HIAA, thus reducing extracellular 5-HT levels. The decrease in 5-HT reduces the activation of neuronal 5-HT1A receptors, possibly inducing the sensation of fatigue. Increases in both proinflammatory cytokines and oxidative and nitrosative stress by activation of microglia via TLR3 could lead to increased IDO activity in microglia, resulting in reduced tryptophan levels, increased kynurenine catabolites, and decreased 5-HT and melatonin synthesis. Quinolinic acid (kynurenine metabolites) is an NAD+ precursor that has neurotoxic properties through binding to the *N*-methyl-d-aspartate (NMDA) receptor, followed by sustained Ca^2+^ influx leading to increased nitrosative stress. The increased oxidative and nitrosative stress generated by all these processes could be exploited by EBV-infected cells for their transformation through viral DNA methylation, preventing their replication and favoring the establishment of different types of latency that allow evasion of the immune system.

## Data Availability

Not applicable.
